# Cortical activation and functional connectivity during locomotion tasks in Parkinson’s disease with freezing of gait

**DOI:** 10.3389/fnagi.2023.1068943

**Published:** 2023-03-08

**Authors:** HongSheng Feng, YanNa Jiang, JinPeng Lin, WenTing Qin, LingJing Jin, Xia Shen

**Affiliations:** ^1^Shanghai YangZhi Rehabilitation Hospital (Shanghai Sunshine Rehabilitation Center), School of Medicine, Tongji University, Shanghai, China; ^2^School of Kinesiology, Shanghai University of Sport, Shanghai, China; ^3^Department of Neurology and Neurological Rehabilitation, Shanghai YangZhi Rehabilitation Hospital (Shanghai Sunshine Rehabilitation Center), School of Medicine, Tongji University, Shanghai, China; ^4^Rehabilitation Medicine Research Center, Shanghai YangZhi Rehabilitation Hospital (Shanghai Sunshine Rehabilitation Center), School of Medicine, Tongji University, Shanghai, China

**Keywords:** Parkinson’s disease, freezing of gait, cerebral haemodynamic, functional connectivity, functional near-infrared spectroscopy

## Abstract

**Background:**

Freezing of gait (FoG) is a severely disabling symptom in Parkinson’s disease (PD). The cortical mechanisms underlying FoG during locomotion tasks have rarely been investigated.

**Objectives:**

We aimed to compare the cerebral haemodynamic response during FoG-prone locomotion tasks in patients with PD and FoG (PD-FoG), patients with PD but without FoG (PD-nFoG), and healthy controls (HCs).

**Methods:**

Twelve PD-FoG patients, 10 PD-nFoG patients, and 12 HCs were included in the study. Locomotion tasks included normal stepping, normal turning and fast turning ranked as three difficulty levels based on kinematic requirements and probability of provoking FoG. During each task, we used functional near-infrared spectroscopy to capture concentration changes of oxygenated haemoglobin (ΔHBO_2_) and deoxygenated haemoglobin (ΔHHB) that reflected cortical activation, and recorded task performance time. The cortical regions of interest (ROIs) were prefrontal cortex (PFC), supplementary motor area (SMA), premotor cortex (PMC), and sensorimotor cortex (SMC). Intra-cortical functional connectivity during each task was estimated based on correlation of ΔHBO_2_ between ROIs. Two-way multivariate ANOVA with task performance time as a covariate was conducted to investigate task and group effects on cerebral haemodynamic responses of ROIs. *Z* statistics of z-scored connectivity between ROIs were used to determine task and group effects on functional connectivity.

**Results:**

PD-FoG patients spent a nearly significant longer time completing locomotion tasks than PD-nFoG patients. Compared with PD-nFoG patients, they showed weaker activation (less ΔHBO_2_) in the PFC and PMC. Compared with HCs, they had comparable ΔHBO_2_ in all ROIs but more negative ΔHHB in the SMC, whereas PD-nFoG showed SMA and PMC hyperactivity but more negative ΔHHB in the SMC. With increased task difficulty, ΔHBO_2_ increased in each ROI except in the PFC. Regarding functional connectivity during normal stepping, PD-FoG patients showed positive and strong PFC-PMC connectivity, in contrast to the negative PFC-PMC connectivity observed in HCs. They also had greater PFC-SMC connectivity than the other groups. However, they exhibited decreased SMA-SMC connectivity when task difficulty increased and had lower SMA-PMC connectivity than HCs during fast turning.

**Conclusion:**

Insufficient compensatory cortical activation and depletion of functional connectivity during complex locomotion in PD-FoG patients could be potential mechanisms underlying FoG.

**Clinical trial registration:**

Chinese clinical trial registry (URL: http://www.chictr.org.cn, registration number: ChiCTR2100042813).

## Highlights

– Increased global cortical activation is elicited by more complex locomotion tasks.
– Inefficient compensatory activation in the PFC, SMA, and PMC during locomotion tasks in PD-FoG patients.
– Unstable functional connectivity during locomotion tasks in PD-FoG patients.

## 1. Introduction

Freezing of gait (FoG) is a severely disabling symptom in Parkinson’s disease (PD) characterized by a brief, episodic absence or a marked reduction of forward progression of the feet during locomotion. It typically occurs when initiating gait or turning (Bloem et al., 2004; Giladi and Nieuwboer, 2008; Nutt et al., 2011). It may directly cause falls and affect the quality of life of patients with PD (Moore et al., 2007; Kerr et al., 2010). More than 60% of patients with PD experience FoG during the disease course (Forsaa et al., 2015).

Multiple neural mechanisms that might underlie FoG have been proposed, such as abnormal gait pattern generation, impairment of automaticity, perceptual malfunction, abnormal coupling of posture with gait, and executive dysfunction (Nutt et al., 2011; Heremans et al., 2013). These mechanisms have been supported by a large body of neural imaging evidence related to structural and functional abnormalities of the brainstem, cerebellum, basal ganglia, and cortical regions in people with PD and FoG (PD-FoG) compared with those without FoG (PD-nFoG) and healthy participants (Bharti et al., 2019). However, to our knowledge, brain function during locomotion in PD-FoG has rarely been investigated. Functional near-infrared spectroscopy (fNIRS) is a non-invasive optical imaging technique that can monitor cerebral haemodynamic responses, which are characterized by changes in the concentration of oxygenated and deoxygenated haemoglobin (ΔHBO_2_ and ΔHHB, respectively) in the brain cortex, by computing the differential density of light between the source and detector optodes. It is increasingly used to analyse patterns of cortical activity during locomotion tasks due to its low susceptibility to motion artefacts (Menant et al., 2020; Lin et al., 2021).

We used fNIRS to investigate the cerebral haemodynamic response during fall-prone locomotion tasks among PD-FoG patients, PD-nFoG patients, and healthy controls (HCs). Multiple cortical regions related to locomotion or FoG were monitored by fNIRS as the regions of interest (ROIs), including the prefrontal cortex (PFC), supplementary motor area (SMA), premotor cortex (PMC), and sensorimotor cortex (SMC) with the primary motor cortex and primary sensory cortex involved (Bharti et al., 2019; Menant et al., 2020). Based on the neurovascular coupling process, (Csipo et al., 2019; Menant et al., 2020) the ΔHBO_2_ and ΔHHB in the ROIs and the correlations of the ΔHBO_2_ between the ROIs were used to reflect cortical activation and connectivity, respectively. Normal stepping, normal turning, and fast turning were set as locomotion test tasks. They were regarded with three difficulty levels based on the kinematic requirements (Lin et al., 2021) and the probability of provoking FoG (Gilat et al., 2015; Mellone et al., 2016). We aimed to clarify the cortical mechanisms underlying FoG by examining the effects of groups and tasks on locomotion-state cortical activation and functional connectivity. Furthermore, we analysed the correlations between cortical activation and clinical features with the secondary aim to explore clinical factors influencing cortical activation. Based on previous evidence, (Nutt et al., 2011; Gilat et al., 2015; Maidan et al., 2015; Belluscio et al., 2019; Bharti et al., 2019) we hypothesised that PD-FoG patients would exhibit a compensatory cortical mechanism during locomotion tasks. However, we also hypothesised that their compensatory capacity would become limited with increasing task difficulty.

## 2. Methods

### 2.1. Participants

For the purpose of this study, we enrolled individuals with PD and age-matched HCs. For participants with PD, the inclusion criteria were those who were having a definite diagnosis of PD, being stably treated with medication, and being able to walk independently for at least 10 m. Those who had nervous system diseases other than PD, cognitive impairment as measured by the Montreal Cognitive Assessment (MoCA, total score <26) (Marinus et al., 2011), or disorders and dysfunctions that affected balance were excluded. The FoG status of PD participants was determined by item III of the Freezing of Gait Questionnaire (FOGQ), with a score of 0 indicating PD-nFoG and a score ≥1 denoting PD-FoG (Giladi et al., 2000; Mancini et al., 2017). For HCs, individuals were eligible if they were 50–80 years (to match those with PD) and were in good health. All participants provided written consent before the experiment. This study was a part of a clinical trial registered on the Chinese Clinical Trial Registry (registration number: ChiCTR2100042813). The study was performed in accordance with the Declaration of Helsinki and was approved by the ethics committee of Shanghai Yangzhi Rehabilitation Hospital (Shanghai Sunshine Rehabilitation Center) (M2017-001/2017-1-23) and the ethics committee of Shanghai Tongji Hospital (2020-062).

### 2.2. Experimental procedures

Personal information, including age, sex, height, weight, static and dynamic balance measured by the One-Leg-Stance (OLS) (Jacobs et al., 2006) and Timed Up and Go tests (TUG) (Mollinedo and Ma Cancela, 2020) respectively, as well as balance confidence quantified by the Activities-specific Balance Confidence Scale (ABC) (Mak and Pang, 2009), were recorded for all participants. Participants with PD were tested during their ‘ON’ medication state. Disease severity was determined using the motor subscale of the Movement Disorders Society Unified Parkinson’s disease Rating Scale (UPDRS-III), (Goetz et al., 2008) and was further categorised by the Hoehn and Yahr Rating Scale. Gait ability was evaluated by the FOGQ, (Scully et al., 2021) and cognitive function by MoCA (Nasreddine et al., 2005).

During the experimental locomotion tasks, participants first stood quietly for 3 min while staring at a ‘cross’ symbol approximately 3 m in front of them and then performed three tasks. These tasks included (1) straight stepping forward and back for three continuous repetitions at a self-selected comfortable pace (normal stepping, NS), (2) pivot turning-in-place and back for three continuous repetitions at a self-selected comfortable pace (normal turning, NT), and (3) pivot turning-in-place and back for three continuous repetitions at fast pace (fast turning, FT). Each task was executed three times. A pseudorandom order of tasks was set to prevent a potential influence of fatigue or learning effects of the later tasks on between-task comparisons. The order was as follows: NS, NT, FT, NT, FT, NS, FT, NS, and NT. The duration of each task was set at 40 s. During these 40 s, once participants heard the instructions (“normal stepping”/“normal turning”/“fast turning”), they performed three repetitions of each task movement as required, and then stood still while staring at the ‘cross’ symbol in front of them until the instruction for next task was given. E-Prime 3.0 (Psychology Software Tools, Inc., Pittsburgh, PA, United States) was used to design the sequence of the tasks and present the cue for each task onset. The performance time of each task was recorded from the starting instruction to the time when participants returned to the initial position after completing three repetitions of each task movement.

**Figure 1:**
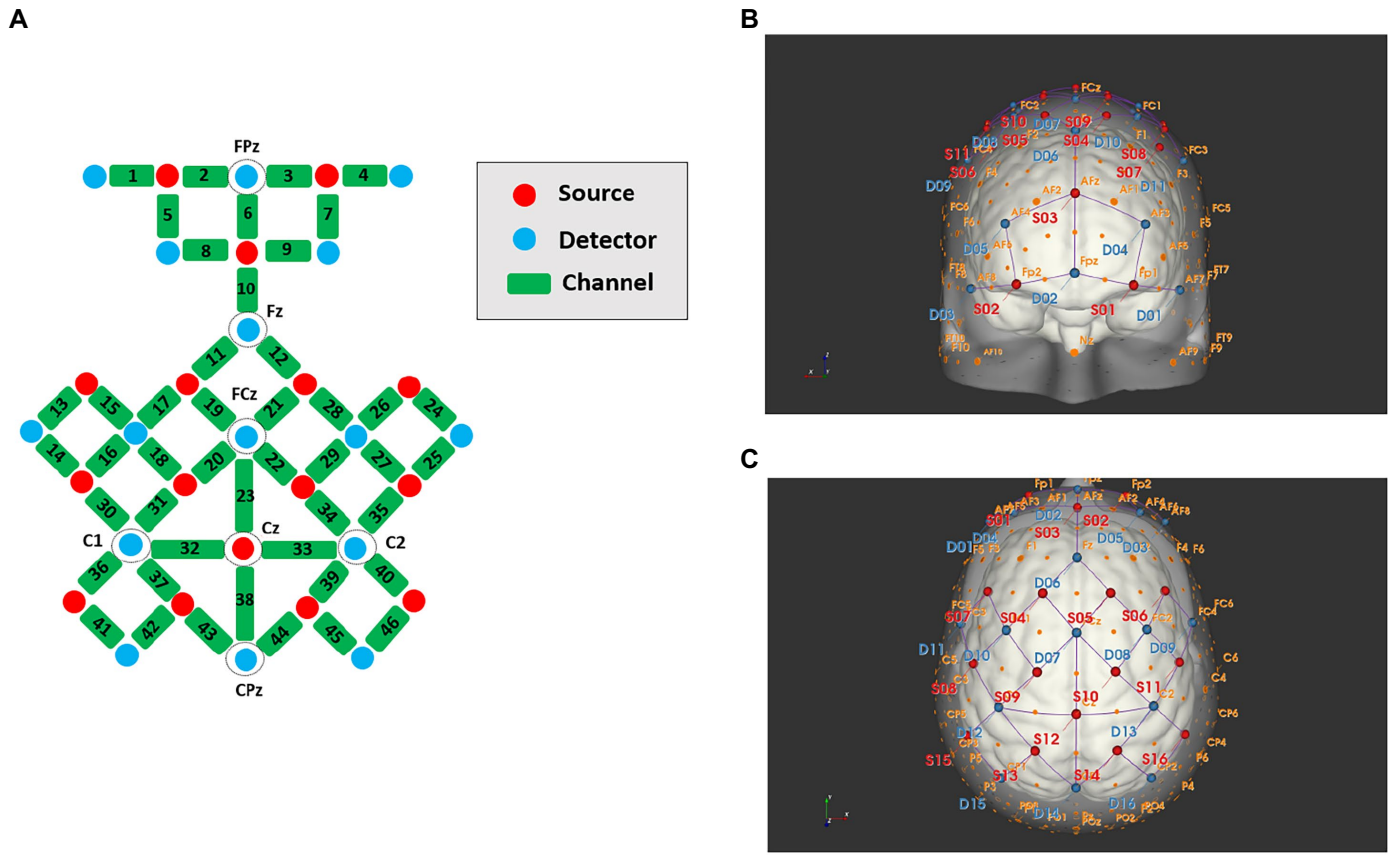
Schematic diagrams of the source, detector, and channel locations, **(A)** channel planimetric map; **(B)** front view; **(C)** plan view.

During locomotion tasks, a portable 16 sources × 16 detectors fNIRS system (NIRSport 2; NIRx Medical Technologies, LLC, Berlin, Germany) was used to measure cortical haemodynamic responses at a sampling rate of 5.0863 Hz. A total of 16 sources and 16 detectors with wavelengths of 760 and 850 nm were symmetrically positioned on each hemisphere with reference to the scalp location, using the method of the international 10–5 system. The arrangement of sources and detectors was composed of 46 channels. Considering that scalp-cortex distance is higher in brain regions near the sagittal midline and frontal pole than in the lateral regions, (Stokes et al., 2005) we set the source and detector approximately 40 mm apart in these regions and approximately 30 mm in other regions. The channel numbers and corresponding anatomical areas for each hemisphere are demonstrated in Figure 1. They covered multiple ROIs, including the PFC (ch1-9), SMA (ch19-23), PMC (ch16-18, 27–35), and SMC (ch41-46) based on the extended international 10–5 head surface-based positioning system (Jurcak et al., 2007).

The fNIRS system was coupled with the E-prime system, with the latter sending a synchronisation signal to the former through their serial ports as soon as the onset cue of each task was presented.

### 2.3. fNIRS data processing

The fNIRS data were analysed using the open-access software Homer 2 within MATLAB (R2020a; Mathworks, Natick, MA, United States). First, the quality of the optical density data of each channel was checked through the quartile coefficient of dispersion. Channels displaying optical density data with quartile coefficient of dispersion <0.4474 were removed. Then, bandpass filtering (low cut-off frequency, 0.01 Hz; high cut-off frequency, 0.2 Hz) was used to eliminate the noise created by heartbeats, breathing, and low-frequency signal drifts (Liu et al., 2018; Lin et al., 2021). Next, the optical density data were converted into ΔHBO_2_ using the modified Beer–Lambert law with constant differential path length factor values of 7.25 for a wavelength of 760 nm and 6.38 for a wavelength of 850 nm. Next, ΔHBO_2_ for each channel of each task was obtained from the blood concentration during the first 20 s of the task subtracted by the mean of a 5s standing phase before the task. The ΔHBO_2_ of three sessions of each task were averaged for further statistical analysis amongst tasks and between participant groups. The ΔHHB data were processed with the same procedure as the ΔHBO_2_ data. The mean ΔHBO_2_ and ΔHHB during the first 20 s of each task were used to reflect the cortical activation associated with each task. There were two reasons for analysing the first 20 s of task duration. First, all subjects completed three repetitions of each task movement within approximately 20 s. Second, 20 s can sufficiently cover the haemodynamic response function of each task (Balters et al., 2021). Besides locomotion tasks, ΔHBO_2_ and ΔHHB during standing were obtained from the average of means of the first 20 s within the first, second, and third minutes of the standing phase.

### 2.4. Statistical analysis

SPSS Statistics version 20 (IBM Corp., Armonk, NY, United States) was used for data analysis. First, a Shapiro–Wilk test was used to check the normality of data of cortical haemodynamic responses in each ROI during each task within each subject group. When normality of data was rejected, clustered boxplot was used to present data outliers for each ROI during each task within each subject group. The data outliers were labelled with corresponding subject codes. Participants whose data presented as outliers more than once in any ROIs during any tasks were excluded from the final analysis. Second, between-group differences in demographic characteristics, and balance function and confidence were examined with a one-way ANOVA for parametric data amongst the three subject groups. An independent *t*-test for parametric data or a Chi-squared (*χ*^2^) test for non-parametric data was performed for comparisons between two subject groups (PD-FoG and PD-nFoG groups). Third, an one-way ANOVA was used to examine the difference in cortical ΔHBO_2_ during standing between the three groups. Fourth, a two-way ANOVA (groups: PD-FoG patients, PD-nFoG patients, and HCs; tasks: NS, NT, and FT) was used to investigate the effects of subject groups and tasks on performance time and ΔHBO_2_ during tasks. The task performance time was analysed using a univariate two-way ANOVA. The ΔHBO_2_ in all ROIs were examined using a multivariate two-way ANOVA, with task performance time as a covariate. If there was a significant group × task interaction effect or group or task effect on ΔHBO_2_ in any ROI, post-hoc between-group or multiple between-task comparisons were conducted. The ΔHHB data were analysed with the same statistical tests described in the third and fourth steps of the ΔHBO_2_ data analysis. Fifth, Pearson correlation tests were used to estimate the functional connectivity between ROIs based on the ΔHBO_2_. The Pearson correlation coefficient was transformed into a z-score with a Fisher transformation, and the obtained z-scores were compared with the Z statistic to determine the differences in functional connectivity between subject groups and between tasks (Bai et al., 2020). Sixth, stepwise multivariate linear regression analyses were used to explore the correlation between clinical features and cortical activation in each ROI during locomotion tasks in the PD-FoG and PD-nFoG groups.

The significance level was set at *p* < 0.05 for all tests. A Bonferroni correction was applied for post-hoc multiple between-task (NS vs. NT vs. FT) and between-groups comparisons (PD-FoG patients vs. PD-nFoG patients vs. HCs) to avoid the inflation of type I errors (*p* < 0.025).

## 3. Results

A total of 28 participants with PD (17 PD-FoG and 11 PD-nFoG patients) and 13 HCs were eligible to participate in this study. After excluding participants with data outliers, 22 participants with PD (12 PD-FoG and 10 PD-nFoG patients) and 12 HCs were included in the final analysis.

PD-FoG patients had lower OLS times than PD-nFoG patients and HCs and had greater disease severity reflected by UPDRS-III and higher FOGQ scores than PD-nFoG patients (*p* < 0.05). Other demographic characteristics were comparable among the subject groups (Table 1).

**Table 1:** Demographic characteristics and clinical features of the participants.

Variables	PD-FOG (*N* = 12)	PD-nFOG (*N* = 10)	HC (*N* = 12)	*p* ^a^
Age (year)	65.7 ± 4.8	68.9 ± 5.5	66 ± 8.5	0.463
Sex (F/M)^	5/7	7/3	8/4	0.32
Body Mass Index (kg/m^2^)	23 ± 2.8	23.5 ± 3.3	23.6 ± 2.3	0.854
OLS time (s)	6.8 ± 6.9^#^	11.7 ± 8.6	17.6 ± 13.6	0.046*
TUG time (s)	11.1 ± 1.5	10.4 ± 1.9	9.9 ± 1.4	0.244
ABC score (0–100)	84.2 ± 11.3	92.3 ± 7.8	92.4 ± 8.3	0.068
PD duration (months)	90.1 ± 47.4	57.2 ± 26	-	0.232
HY stage (0–5)^	2 (2–3)	2 (2–3)	-	0.189
UPDRS-III (0–132)	37.4 ± 11.0	25.7 ± 8.5		0.012*
FOGQ (0–24)	9.3 ± 4.3	1.8 ± 1.4	-	0.000*
MoCA (0–30)	27.5 ± 0.9	27.8 ± 1.1	-	0.505

PD-FoG, Parkinson’s disease with freezing of gait; PD-nFoG, Parkinson’s disease without freezing of gait; HC, healthy control; OLS, One-Leg Stance test; TUG, Timed Up and Go test; ABC, activities-specific balance confidence scale; PD, Parkinson’s disease; HY, Hoehn and Yahr Rating Scale; UPDRS, unified Parkinson’s disease rating scale; FOGQ, freezing of gait questionnaire; MoCA, Montreal Cognitive Assessment. ^a^All *p* values resulted from one-way analysis of variance for the three groups or independent *t*-tests for two groups, except those for sex and HY stage marked with ^, which were analyzed by *χ*^2^ test. ^#^OLS time of PD-FoG was shorter than that of PD-nFoG and HC in contrast tests after one-way analysis of variance for the three groups. *p < 0.05.

### 3.1. Cortical activation in ROIs

Each group showed comparable ΔHBO_2_ and ΔHHB in each ROI during the standing phase (*p* > 0.05; Table 2). Task performance time showed significant task effects (*p* < 0.05; Table 3). FT needed a shorter time than NT and NS (*p* < 0.025), while there was no difference between NT and NS. Regarding the group effect, PD-FoG patients showed a longer time, which reached statistical near-significance, than PD-nFoG patients (*p* = 0.055). However, there was no difference between PD-nFoG patients and HCs. Using a two-way multivariate ANOVA with task performance time as a covariate, we found significant general group and task effects on ΔHBO_2_ in the ROIs (*p* < 0.05), but there were no significant interaction effects of the group and the task (*p* > 0.05). Among the ROIs, group effects reached significance only in the PFC, PMC, and SMA (*p* < 0.05). A *post-hoc* multiple between-groups comparison showed that PD-FoG patients had significantly less ΔHBO_2_ in the PFC and PMC than did PD-nFoG patients (*p* < 0.025). PD-nFoG patients but not PD-FoG patients had significantly higher ΔHBO_2_ in the SMA (*p* < 0.025) and near-significantly higher in the PMC (*p* = 0.030) than HCs. Task effects reached significance in all ROIs except in the PFC (*p* < 0.05) showing larger ΔHBO_2_ as the difficulty of tasks increased. Results of post-hoc between-task comparison of ΔHBO_2_ are shown in Table 4. Unlike ΔHBO_2_, the ΔHHB was overall stable across ROIs and among groups, except for a larger decrease in the SMC of PD-FoG and PD-nFoG patients than in HCs (*p* = 0.087 and *p* = 0.001, respectively; Table 4).

**Table 2:** One-way ANOVA results for haemodynamic responses of ROIs during the standing phase among the PD-FOG, PD-nFOG, and HC groups.

Variables	ROIs	PD-FOG (*N* = 12)	PD-nFOG (*N* = 10)	HC (*N* = 12)	*p*
∆HBO_2_	PFC	−0.02 ± 0.04	0.02 ± 0.11	0.02 ± 0.08	0.417
	SMA	0.03 ± 0.08	0.02 ± 0.06	0.00 ± 0.07	0.627
	PMC	0.01 ± 0.05	0.03 ± 0.07	−0.01 ± 0.05	0.244
	SMC	0.02 ± 0.06	0.05 ± 0.07	−0.01 ± 0.07	0.221
∆HBB	PFC	0.01 ± 0.02	0.00 ± 0.02	−0.02 ± 0.05	0.093
	SMA	0.01 ± 0.02	0.00 ± 0.01	0.01 ± 0.03	0.306
	PMC	0.01 ± 0.03	0.00 ± 0.01	0.00 ± 0.02	0.432
	SMC	0.00 ± 0.02	−0.01 ± 0.03	−0.01 ± 0.02	0.320

Values are presented as mean ± standard deviation (μmol/L). ∆HBO2, change of oxygenated haemoglobin; ∆HBB, change of deoxygenated haemoglobin; ROI, region of interest; PD, Parkinson’s disease; HC, healthy control; PD-FoG, Parkinson’s disease with freezing of gait; PD-nFoG, Parkinson’s disease without freezing of gait; PFC, prefrontal cortex; SMA, supplementary motor area; PMC, premotor-motor cortex; SMC, sensorimotor cortex.

**Table 3:** Two-way ANOVA results of task performance time during different tasks among the PD-FOG, PD-nFOG, and HC groups.

Task performance time	PD-FOG (*N* = 12)	PD-nFOG (*N* = 10)	HC (*N* = 12)	Group effects	PD-FOG vs. PD-nFOG	PD-FOG vs. HC	PD-nFOG vs. HC	Task effects	NS vs. NT	NT vs. FT	NS vs. FT
(Seconds)		Mean ± SD		*p* values							
NS	9.9 ± 1.1	9.4 ± 1.6	10.4 ± 2.5								
NT	10.5 ± 2.2	9.5 ± 1.5	9.1 ± 1.8	0.116	0.055	0.108	0.693	0.000*	0.724	0.000*	0.000*
FT	8.0 ± 1.7	6.9 ± 1.1	6.8 ± 1.5								

Group × task effects of ANOVA test: *p* = 0.509. *p < 0.05 for group effects and task effects; *p < 0.025 for between-task comparisons (NS vs. NT, NT vs. FT, and NS vs. FT). ANOVA, analysis of variance; PD, Parkinson’s disease; HCs, healthy controls; PD-FoG, Parkinson’s disease with freezing of gait; PD-nFoG, Parkinson’s disease without freezing of gait; SD, standard deviation; NS, normal stepping task; NT, normal turning task; FT, fast turning task.

**Table 4:** Two-way ANOVA results of haemodynamic responses of ROIs during different tasks among the PD-FOG, PD-nFOG, and HC groups.

	PD-FOG (*N* = 12)	PD-nFOG (*N* = 10)	HC (*N* = 12)	Group effects	PD-FOG vs. PD-nFOG	PD-FOG vs. HC	PD-nFOG vs. HC	Task effects	NS vs. NT	NT vs. FT	NS vs. FT	Task performance time
	Mean ± SD			*p* values								
**∆HBO**_**2**_ **in each ROI (μmol/L)**													
PFC	NS	0.07 ± 0.28	0.38 ± 0.49	0.3 ± 0.35									
	NT	0.21 ± 0.32	0.33 ± 0.16	0.21 ± 0.25	0.034*	0.009*	0.199	0.150	0.249	0.948	0.136	0.128	0.01*
	FT	0.26 ± 0.24	0.31 ± 0.27	0.22 ± 0.23									
SMA	NS	0.00 ± 0.21	0.14 ± 0.15	−0.07 ± 0.19									
	NT	0.13 ± 0.17	0.14 ± 0.11	−0.03 ± 0.3	0.027*	0.161	0.182	0.007*	0.041*	0.232	0.117	0.012*	0.186
	FT	0.12 ± 0.15	0.15 ± 0.14	0.12 ± 0.3									
PMC	NS	0.00 ± 0.21	0.15 ± 0.13	−0.04 ± 0.26									
	NT	0.11 ± 0.14	0.21 ± 0.11	0.10 ± 0.23	0.025*	0.010*	0.633	0.030#	0.003*	0.028#	0.123	0.001*	0.038*
	FT	0.12 ± 0.12	0.18 ± 0.1	0.19 ± 0.31									
SMC	NS	−0.02 ± 0.3	0.07 ± 0.25	−0.06 ± 0.24									
	NT	0.13 ± 0.22	0.13 ± 0.11	0.1 ± 0.34	0.698	0.448	0.961	0.468	0.001*	0.028#	0.043#	0.000*	0.012*
	FT	0.17 ± 0.16	0.14 ± 0.13	0.19 ± 0.28									
Overall ROIs					0.018*				0.028*				
**∆HBB in each ROI (μmol/L)**													
PFC	NS	0.00 ± 0.06	0.04 ± 0.22	0.00 ± 0.12									
	NT	0.01 ± 0.07	−0.02 ± 0.06	−0.02 ± 0.09	0.625	0.908	0.372	0.456	0.440	0.587	0.201	0.422	0.259
	FT	−0.01 ± 0.09	−0.03 ± 0.06	0.1 ± 0.3									
SMA	NS	−0.02 ± 0.04	−0.05 ± 0.06	−0.05 ± 0.16									
	NT	−0.02 ± 0.05	−0.05 ± 0.04	−0.08 ± 0.15	0.128	0.147	0.052	0.680	0.468	0.701	0.362	0.224	0.111
	FT	−0.03 ± 0.08	−0.06 ± 0.03	−0.07 ± 0.17									
PMC	NS	−0.02 ± 0.06	−0.05 ± 0.07	−0.01 ± 0.08									
	NT	−0.02 ± 0.05	−0.03 ± 0.07	−0.02 ± 0.06	0.194	0.403	0.322	0.072	0.897	0.904	0.719	0.648	0.44
	FT	−0.03 ± 0.06	−0.06 ± 0.07	0.02 ± 0.23									
SMC	NS	−0.04 ± 0.05	−0.09 ± 0.07	−0.04 ± 0.09									
	NT	−0.04 ± 0.05	−0.06 ± 0.07	0.02 ± 0.07	0.006*	0.113	0.087#	0.001*	0.281	0.113	0.433	0.568	0.324
	FT	−0.05 ± 0.06	−0.09 ± 0.05	−0.03 ± 0.14									
Overall ROIs					0.000*				0.337				

Group × task effects of multivariate ANOVA test of ∆HBO_2_ and ∆HBB: *p* = 0.885 and 0.847, respectively. *p < 0.05 for group and task effects; *p < 0.025 for between-subject (PD-FoG vs. PD-nFoG patients, PD-nFoG patients vs. HCs, and PD-FoG patients vs. HCs) and between-task comparisons (NS vs. NT, NT vs. FT, and NS vs. FT); #*p* < 0.1, nearly significant. ANOVA, two-way analysis of variance; ∆HBO_2_, change of oxygenated haemoglobin concentration; ∆HBB, change of deoxygenated haemoglobin concentration; ROI, region of interest; PD-FoG, Parkinson’s disease with freezing of gait; PD-nFoG, Parkinson’s disease without freezing of gait; HC, healthy control; SD, standard deviation; PFC, prefrontal cortex; SMA, supplementary motor area; PMC, premotor-motor cortex; SMC, sensorimotor cortex; NS, normal stepping task; NT, normal turning task; FT, fast turning task.

### 3.2. Functional connectivity between ROIs

**Figure 2:**
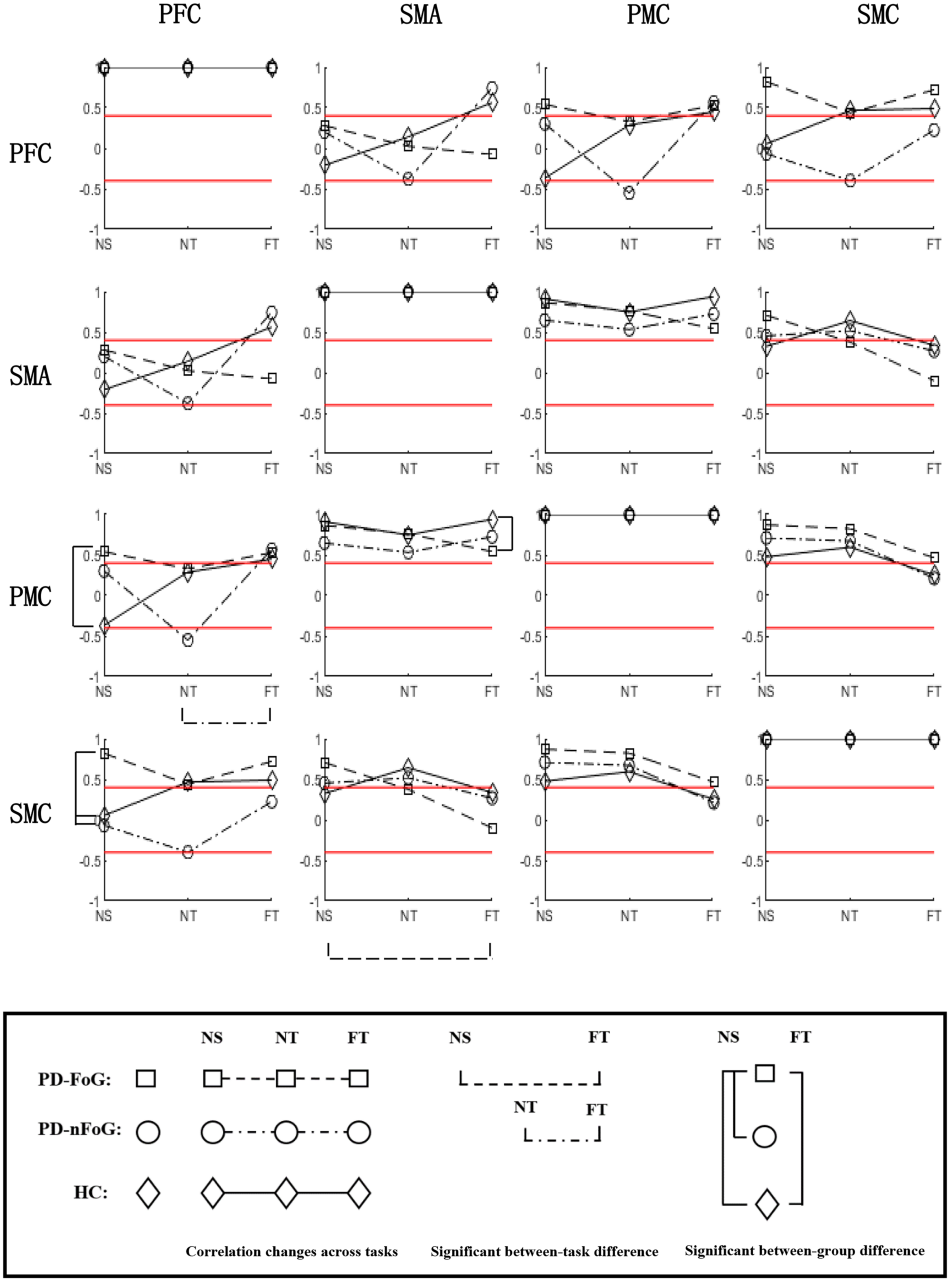
Correlation matrix of ∆HBO_2_ between ROIs during different tasks in each participant group. For each subplot, the values of the y-axis are the correlation coefficients of ΔHBO_2_ between ROIs during each task in each participant group. On the x-axis, NS, NT, and FT represent the three locomotion tasks, namely normal stepping, normal turning, and fast turning, respectively. The icons □, ○, and ◇ in each subplot denote the correlation coefficients of ΔHBO_2_ between ROIs in PD-FoG patients, PD-nFoG patients, and HCs, respectively. The dashed, dash-dotted, and solid lines in each subplot denote the change of correlation coefficients of ΔHBO_2_ between ROIs from NS to NT to FT in PD-FoG patients, PD-nFoG patients, and HCs, respectively. Red lines signify a correlation coefficient value at 0.4 or-0.4, which is the threshold for moderate correlation (Evans, 1996). Vertical square brackets indicate significant between-group difference in z-scores of correlations coefficients during a task (*p* < 0.05). The type and the x-axis position of the icons marked by brackets indicate the corresponding groups and tasks explained above. Horizontal square brackets indicate significant between-task difference in the z-scores of the correlation coefficients within a participant group. The dashed, dash-dotted lines of the horizontal square brackets indicate PD-FoG patients and PD-nFoG patients, respectively. PFC, prefrontal cortex; PMC, premotor-motor cortex; SMA, supplementary motor area; SMC, sensorimotor cortex; ROI, region of interest.

PD-FoG patients showed positive strong PFC-PMC connectivity, opposite of the negative PFC-PMC connectivity observed in HCs. They also had stronger PFC-SMC connectivity compared to other groups, during NS. During FT, they had weaker SMA-PMC connectivity than HCs. In PD-FoG patients, when task difficulty increased, the connectivity between SMA-SMC significantly decreased, while a decreasing trend was observed between the other ROIs. Conversely, an increasing trend was observed in HCs, and an unstable trend was observed in PD-nFoG patients (Figure 2).

### 3.3. Correlations between clinical features and cortical activation

**Figure 3:**
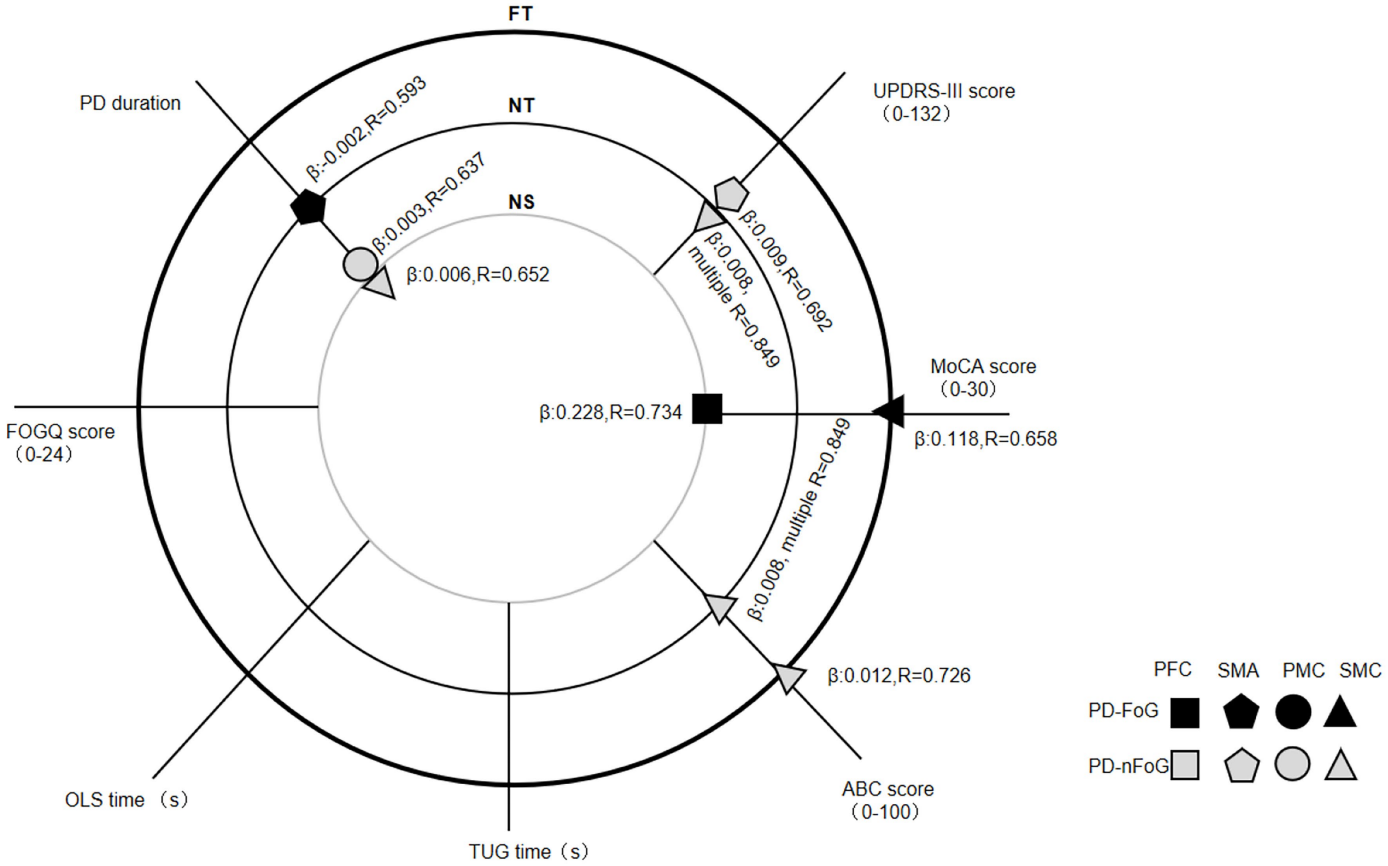
Regression analysis results on the correlation between clinical features and cortical activation in each ROI during different locomotion tasks within each PD patient group. The three big circles with a thin grey line (inner), thin black line (middle), and bold black line (outer) indicate locomotion tasks of normal stepping (NS), normal turning (NT) and fast turning (FT), respectively. The small icons with shape of a square, pentagon, circle and triangle denote the cortical activation in PFC, SMA, PMC and SMC, respectively. The black and grey icons denote the groups of PD-FoG patients and PD-nFoG patients, respectively. An icon with information of ROI and group, marking at the cross of a clinical feature line, and a task circle denoted that the clinical feature is a significant determinant of the activation in the specific ROI during the specific task of the specific group. For each significant determinant, the coefficient in estimated regression equation (*β*) and the strength of the linear relationship (R) are reported. PD-FoG, Parkinson’s disease with freezing of gait; PD-nFoG, Parkinson’s disease without freezing of gait; PFC, prefrontal cortex; PMC, premotor-motor cortex; SMA, supplementary motor area; SMC, sensorimotor cortex; ROI, region of interest.

Regression analysis on the correlation between clinical features and cortical activation identified four clinical features independently predicting cortical activation specific to some ROIs, locomotion tasks and subject groups. PD duration was positively correlated with PMC [the coefficient in estimated regression equation *β* (95% confidence interval [CI]): 0.003 (0.000, 0.006), the strength of the linear relationship (*R* = 0.637, adjusted *R*^2^ = 0.334) and SMC activation [*β* (95% CI): 0.006 (0.000, 0.012), *R* = 0.652, adjusted *R*^2^ = 0.353] during NS in PD-nFoG patients. Conversely, it was negatively correlated with SMA activation during NT in PD-FoG patients [*β* (95% CI): −0.002 (−0.004, 0.000), *R* = 0.593, adjusted *R*^2^ = 0.353]. UPDRS-III scores were positively correlated with SMA [*β* (95% CI): 0.009 (0.001, 0.016), *R* = 0.692, adjusted *R*^2^ = 0.413] and SMC activation during NT [*β* (95% CI): 0.008 (0.001, 0.014), multiple *R* = 0.849, adjusted *R*^2^ = 0.641 for the model combining UPDRS-III with ABC score] in PD-nFoG patients. The MoCA score was positively correlated with PFC activation during NS [*β* (95% CI): 0.228 (0.080, 0.337), *R* = 0.734, adjusted *R*^2^ = 0.493] and SMC activation during FT [*β* (95% CI): 0.118 (0.023, 0.213), *R* = 0.658, adjusted *R*^2^ = 0.376] in PD-FoG patients. The ABC score showed positive correlation with SMC activation during NT [*β* (95% CI): 0.008 (0.001, 0.014), multiple *R* = 0.849, adjusted *R*^2^ = 0.641 for the model combining UPDRS-III with ABC score] and FT [*β* (95% CI): 0.012 (0.003, 0.021), *R* = 0.726, adjusted *R*^2^ = 0.468)] in PD-nFoG patients. The FOGQ, TUG time and OLS time showed no correlation with cortical activation in neither PD-FoG nor PD-nFoG patients (Figure 3).

## 4. Discussion

To our knowledge, this is the first study to explore the neural mechanisms underlying FoG by examining cerebral haemodynamic responses in multiple cortical ROIs during FoG-prone locomotion tasks using fNIRS in PD-FoG and PD-nFoG patients, and HCs. The results partially support our hypothesis that PD-FoG patients compensate during locomotion by increasing PFC-SMC connectivity and not by boosting overall cortical activation. Furthermore, the results indicated that PD-FoG patients show deficits in the compensatory mechanism with increased task difficulty.

### 4.1. Cortical activation in ROIs

Based on evidence from neuroimaging studies, two distinct supraspinal locomotor networks (direct and indirect) have been suggested to be responsible for the automatic and executive control of locomotion, respectively (la Fougère et al., 2010; Hamacher et al., 2015; Herold et al., 2017). The direct locomotor network, which automatically controls stereotype gait, runs directly from the primary motor cortex to the spinal cord paced by the rhythm from cerebellar locomotor region (Gilat et al., 2017). The indirect locomotor network, which controls modulated gait *via* executive function, (Zwergal et al., 2013) transmits neuronal commands from the frontal cortex to the brainstem locomotor regions *via* the basal ganglia. Evidence shows dysfunction of direct locomotor network and compensatory activation of indirect locomotor network in PD patients during virtual reality gait, who demonstrate obvious step variability, a sign of reduced gait automaticity (Zwergal et al., 2013). By examining multiple cortical ROIs we could explore the cortical mechanisms underlying FoG in greater depth compared to previous fNIRS studies that only considered sole regions (Stuart et al., 2018; Lin et al., 2021).

The PFC has been extensively examined, often as a single ROI in previous fNIRS studies on locomotion-state brain activation of PD (Stuart et al., 2018; Lin et al., 2021). Notably, among the multiple ROIs we examined in the present study, the PFC showed a much higher activation during locomotion than other regions in both PD patients and HCs. Therefore, PFC activation could be a preferential attempt to retain more attention or consciousness during executive function to improve locomotion performance (Burton and Sinclair, 2000; Stoeckel et al., 2003; Yogev-Seligmann et al., 2008). Previous studies have observed that compared to HCs, PD patients in either “ON” or “OFF” medication states show larger PFC activation during regular walking tasks, which may be a compensatory strategy in response to impaired gait automaticity (Maidan et al., 2016; Orcioli-Silva et al., 2020, 2021). However, the compensatory PFC capacity in PD patients during ‘OFF’ state has been shown to be insufficient to cope with complex locomotion tasks such as dual-task walking and walking with obstacle avoidance (Orcioli-Silva et al., 2020, 2021). This could result from an excessive inhibitory output from the basal ganglia to the frontal cortex due to dopamine insufficiency (Obeso et al., 2008). Dopaminergic medication has proven effective in correcting the basal ganglia circuit and thus maintaining the compensatory capacity of the PFC allowing optimal performance of complex tasks in PD patients (Orcioli-Silva et al., 2020; Dagan et al., 2021; Orcioli-Silva et al., 2021). All PD patients in the present study were examined after medication intake. However, PD-FoG patients showed less PFC activation during locomotion tasks than PD-nFoG patients, indicating a lower PFC compensatory capacity in PD-FoG patients. It should be noted that the PFC attentional resource has limited capacity even in healthy people. Here, we observed that with increased task difficulty, the PFC was the only region without additional increase in activation especially in PD-nFoG patients and HCs. The PFC activation elicited by locomotion tasks in PD patients and HCs in this study was comparable with that during dual-task walking in PD patients (0.29 μmol/l) and older adults (0.31–0.34 μmol/l) in previous studies (Maidan et al., 2016; Mirelman et al., 2017). This might indicate that the locomotion tasks in our study, including NS, NT, and FT, might have exceeded the maximum capacity of the PFC, possibly due to the repetitive initiation and termination involved in each task requiring the same high level of attention as that required in dual-task walking. Therefore, as a result of limited resources in the PFC, the SMA and PMC followed in responding to the increased task difficulty (Zhang et al., 2022).

Accumulating evidence suggests that the SMA is a key region involved in coupling of posture with gait (Nutt et al., 2011) that triggers anticipatory postural control through dense projections to the brainstem reticular formation and determines the time of gait initiation by interaction with the basal ganglia and cerebellum locomotor region (Herold et al., 2017; Takakusaki et al., 2017). Therefore, dysfunction of the SMA has been identified as the crucial resource of FoG induced by abnormal coupling of posture with gait. Reduction in SMA gray mass (Vastik et al., 2017) and reduced activation and connectivity with the basal ganglia at resting state (Matar et al., 2019) and during imagined or virtual reality locomotion tasks (Shine et al., 2013; Peterson et al., 2014) have been observed in PD-FoG patients compared to PD-nFoG patients. PD patients with SMA dysfunction have demonstrated responsiveness to dopaminergic medication, which is thought to enhance SMA activation and connectivity with the basal ganglia, thereby improving locomotion performance (Almeida et al., 2007; Matar et al., 2019). In this study, after medication intake, PD-nFoG patients showed comparable locomotion performance with HCs. However, they also exhibited SMA hyperactivation during locomotion tasks when compared with HCs. This might be a compensatory strategy of shifting to executive control for coupling posture and gait and in response to impaired gait automaticity, similar to the PFC compensation mentioned previously. PD-FoG patients demonstrated comparable activation of the SMA with a trend of poorer performance in locomotion tasks compared to HCs. These results imply that PD-FoG patients lack the SMA compensatory mechanism needed to ensure good locomotion performance.

The PMC plays important roles in motor programming and modulation, especially for externally cued movements, (Wessel et al., 1997) *via* rich cortical networks that are interlinked with the prefrontal cortex, parietal cortex and motor cortex (Hoshi and Tanji, 2007; Herz et al., 2014). Patients with PD exhibit hyperactivation of the PMC during finger movement tasks, (Haslinger et al., 2001; Hughes et al., 2010) and increased functional connectivity between the PMC and the primary motor cortex at resting state, (Hao et al., 2020) when compared with HCs. PMC hyperactivation has been suggested as a strategy to compensate for the defective function of the SMA and to prevent improperly timed movement initiation (Hao et al., 2020). The PMC hyperactivation in PD patients can be attenuated to some extent with levodopa medication but it is still present unlike in HCs (Haslinger et al., 2001; Hughes et al., 2010). We found PMC hyperactivation in PD-nFoG patients relative to HCs but not in PD-FoG. Furthermore, we observed PMC hypoactivity during locomotion in PD-FoG patients compared to PD-nFoG patients. These results imply that PD-FoG patients also lack the PMC compensatory mechanism needed to ensure good locomotion performance.

Moreover, with increased task difficulty, increased activation of the SMA and PMC was observed during FT compared to NS. This finding suggests that SMA and PMC activation is specific to task mode and speed, which might help explain why FoG is induced by turning, especially at a fast speed (Gilat et al., 2015; Mellone et al., 2016) whilst also providing evidence for designing exercise training with optimal effect on neural modulation. Furthermore, the SMA was the only region where the degree of activation during locomotion had no correlation with task performance time. This finding may be attributed to the role of the SMA in programming anticipatory postural control to facilitate task initiation, which correlates with the repetition but not with the duration of a task, despite the task’s difficulty level (Herold et al., 2017; Takakusaki et al., 2017).

The SMC, which includes the primary sensory and motor areas of the brain, has been well-known to have a major role in real movement execution *via* direct automatic locomotor control networks (la Fougère et al., 2010; Takakusaki et al., 2017). A previous resting-state functional magnetic resonance imaging study reported SMC hypoactivity in *de novo* PD, (Tessa et al., 2012) but also showed hyperactivity in common PD compared with HC. This hyperactivity is believed to reflect compensatory cortical reorganisation (Wu et al., 2009). Our findings revealed that with increased task difficulty, ΔHBO_2_ in the SMC increased but without any observable between-group differences. The ΔHHB in the SMC did not change with increased task difficulty. However, it decreased during locomotion tasks in PD patients, but not in HCs. A decrease in ΔHHB with comparable ΔHBO_2_ indicates oxygen hypometabolism, meaning lower cerebral metabolic rate of oxygen than required for normal cerebral blood flow (Nakamura et al., 2020). Oxygen hypometabolism has been reported in multiple cortical regions of patients with corticobasal degeneration, (Sawle et al., 1991) suggesting microvascular pathology (Dalby et al., 2021). To our knowledge, we are the first to report oxygen hypometabolism in the SMC of PD patients with and without FoG, which could be of interest to researchers studying oxygen metabolism or PD management. In summary, insufficient compensatory capacity of the PFC, SMA and PMC during locomotion could be underlying FoG.

### 4.2. Functional connectivity between ROIs

Besides the direct and indirect top-down locomotor networks for locomotion control, intracortical networks are important to optimize resources, for guaranteeing accuracy of motor output (Wu et al., 2011). Evidence shows that PD patients have a generally lower intracortical connectivity than HCs, but dopaminergic medication has been shown to be effective in improving connectivity (Herz et al., 2014; Nettersheim et al., 2019). In this study, PD-FoG patients in ‘ON’ medication state showed more PFC-SMC connectivity than HCs and PD-nFoG patients. This indicates that in PD-FoG patients indirect locomotor networks might be activated and coupled with the direct locomotor networks for controlling a simple task, but that might not be necessary in HCs or PD-nFoG patients (Maidan et al., 2016; Orcioli-Silva et al., 2020, 2021). This could be a compensatory mechanism for the impaired locomotion automaticity in PD-FoG patients. Moreover, we observed that PD-FoG patients had positive strong PFC-PMC connectivity, which was in contrast with the observed negative connectivity in HCs. Having similar functions in motor programming and modulation as the SMA, the PMC is usually activated *via* its specific networks to supplement SMA function or to compensate for SMA dysfunction and thus guarantee accuracy of motor output (Hoshi and Tanji, 2007; Herz et al., 2014). The PFC-PMC connectivity is usually observed after the PFC-SMA connectivity during increased task difficulty (Wu et al., 2011; Nettersheim et al., 2019). The observed negative PFC-PMC connectivity in HCs could be a sign of inhibition, which leads to optimization of cortical resources. The earlier occurrence of the compensatory PFC-PMC connectivity in PD-FoG patients but not in HCs would imply a dysfunction of the SMA or an impaired PFC-SMA connectivity in these patients.

In PD-FoG patients the strength of functional connectivity originating from the PFC decreased with increased task difficulty. Conversely, in HCs it gradually increased. Decreased connectivity typically signals depletion of brain resources needed for regulating interregional information transmission (Peng et al., 2021). Furthermore, with increased task difficulty, PD-FoG patients demonstrated decreased functional connectivity between the SMA and SMC, which implied a decoupling between the two areas, possibly due to the depletion of SMA capacity. Functional connectivity between the PMC, SMA and SMC was relatively stable during different locomotion tasks and within subject groups, possibly because they are spatially adjacent and key regions of the action-execution network (Bharti et al., 2019).

In summary, PD-FoG patients showed increased intracortical connectivity, which might have led to the activation of indirect locomotor networks to cope with NS. However, the observed increase in intracortical connectivity was not sustainable to meet the requirements of FT.

### 4.3. Correlations between clinical features and cortical activation

Regarding clinical features and cortical activation, it is worth noting that for PD patients, the balance and gait features measured by FOGQ, TUG time and OLS time were not identified as the independent factor predicting cortical activation in some of the ROIs during NS, NT or FT. Although they did show some correlation with cortical activation through bivariate correlation analysis, it was weaker than other disease-specific, cognitive and psychological features. Therefore, they were excluded from the final regression model. It might be explainable since the disease-specific, cognitive and psychological features, resulting from the underlying neuropathology, could directly affect cortical activation during locomotion; however, the difference in manners and time between tasks of clinical balance and gait tests and experimental locomotion tasks might make their association relatively weaker. Though, real-time task performance might have a direct and strong correlation with cortical activation during tasks. This notion is supported by previous findings and is consistent with our finding of significant association between task performance time and cortical activation in most ROIs. Belluscio et al. examined cortical activation during NS and dual-task turning in PD patients, and found that PFC activation was strongly correlated with FOG-ratio during NS, but that it correlated less with disease-specific and cognitive-specific features in PD-FoG patients (Belluscio et al., 2019).

In PD-nFoG patients, PD duration was positively correlated with PMC and SMC activation during NS and the UPDRS-III was positively correlated with SMA and SMC activation during NT. However, in PD-FoG patients, PD duration was negatively correlated with SMA activation during NT. This relationship indicates that as the disease progresses, PD-nFoG patients use the compensatory capacity of the PMC, SMA and SMC, but PD-FoG patients exhibit deterioration of SMA function without compensation. This indication was supported by our findings on cortical activation and the relevant fore-mentioned evidence (Haslinger et al., 2001; Wu et al., 2009; Hughes et al., 2010; Shine et al., 2013; Peterson et al., 2014).

The MoCA score was positively correlated with PFC activation during NS and with SMC activation during FT only in PD-FoG patients. This association might indicate that cognitive impairment deteriorates the compensatory capacity of PFC and SMC function in PD-FoG patients. Piramide et al. found an association between reduced recruitment of the fronto-striatal circuit measured by functional magnetic resonance imaging and poor cognitive function measured by visuospatial and executive function tests (Piramide et al., 2020). These findings support the hypothesis of cognitive impairment, particularly executive dysfunction, being one of the potential neural mechanisms underlying the development of FOG (Nutt et al., 2011; Heremans et al., 2013). The positive association between ABC scores and SMC activation during NT and FT in PD-nFoG patients could explain the phenomenon where positive emotion facilitates gait performance, which is not prominent in PD-FoG patients (Takakusaki, 2013). In summary, SMA and PFC function in PD-FoG patients deteriorates as the disease and the cognitive impairment progresses.

### 4.4. Limitations

This study had several limitations. First, the sample size was small, which decreased the statistical power of the study, reduced the representability of patients with PD and thus restricted the applicability of the results to all PD patients. Participants with PD in our study were at Hoehn-Yahr stage of 2–3, and their balance ability and confidence, with the exception of OLS time, were still comparable with those of HCs. Therefore, the present results are only applicable to patients with PD at the mild-to-moderate stage without obvious balance disorders. Second, participants with PD were tested during their ‘ON’ medication phase. Medication intake has been found to restore cortical capacity in PD patients to some extent (Haslinger et al., 2001; Almeida et al., 2007; Hughes et al., 2010; Herz et al., 2014; Maidan et al., 2016; Matar et al., 2019; Nettersheim et al., 2019; Orcioli-Silva et al., 2020; Dagan et al., 2021; Orcioli-Silva et al., 2021). Thus, it might have impeded exploring the original mechanisms underlying FoG. The comparison between our findings and previous findings in PD patients during their ‘ON’ medication phase should be prudently understood. Third, because of the cable length of the fNIRS equipment, turning-in-place was designed as an experimental locomotion task. However, the stepping tasks were not sufficiently difficult to trigger FoG, although the patients with PD reported having FoG experiences with different degrees in daily life in the FoGQ questionnaire. Since FT is a more FoG-prone activity, the neural capability limits during FT found in our study could be the mechanism underlying FoG. Additional studies with experiments that can trigger FoG are needed to explore the mechanisms underlying this phenomenon further.

In conclusion, with increased task difficulty, there is increased cortical activation in most ROIs during locomotion in both PD patients and HCs. PD-FoG patients preserve a certain degree of cortical ability to respond to the difficulty level of locomotion tasks. However, they show insufficient cortical activation in the PFC, SMA and PMC. They show earlier activation of the executive control networks during a simple task, but lack stabilising intracortical connectivity with increased task difficulty. The results of this study might be useful in explaining the mechanism underlying FoG and might serve as a guide to design optimal exercise therapies or neural modulation protocols to better manage gait disturbance in PD.

## Data availability statement

The original contributions presented in the study are included in the article/supplementary material, further inquiries can be directed to the corresponding author.

## Ethics statement

The studies involving human participants were reviewed and approved by Shanghai Yangzhi Rehabilitation Hospital (Shanghai Sunshine Rehabilitation Center) (M2017-001/2017-1-23) and the ethics committee of Shanghai Tongji Hospital (2020-062). The patients/participants provided their written informed consent to participate in this study.

## Author contributions

HF: investigation, formal analysis, and writing – original draft. YJ: investigation and data curation. JL and WQ: investigation. LJ: resources, project administration, and writing – review and editing. XS: conceptualization, methodology, resources, supervision, funding acquisition, and writing – review and editing. All authors contributed to the article and approved the submitted version.

## Funding

This work was supported by the National Natural Science Foundation of China [grant number 81802240].

## Conflict of interest

The authors declare that the research was conducted in the absence of any commercial or financial relationships that could be construed as a potential conflict of interest.

## Publisher’s note

All claims expressed in this article are solely those of the authors and do not necessarily represent those of their affiliated organizations, or those of the publisher, the editors and the reviewers. Any product that may be evaluated in this article, or claim that may be made by its manufacturer, is not guaranteed or endorsed by the publisher.

## Abbreviations

FoG: Freezing of gait
PD: Parkinson’s disease
PD-nFoG: Parkinson’s disease without freezing of gait
fNIRS: Functional near-infrared spectroscopy
ΔHBO_2_: Changes in the concentration of oxygenated haemoglobin
ΔHHB: Changes in the concentration of deoxygenated haemoglobin
HCs: Healthy controls
ROI: Regions of interest
PFC: Prefrontal cortex
PMC: Premotor-motor cortex
SMA: Supplementary motor area
SMC: Sensorimotor cortex
MoCA: Montreal Cognitive Assessment
FOGQ: Freezing of Gait Questionnaire
OLS: One-Leg-Stance tests
TUG: Timed Up and Go tests
ABC: Activities-specific Balance Confidence Scale
UPDRS: Unified Parkinson’s disease Rating Scale
NS: Normal stepping
NT: Normal turning
FT: Fast turning
